# *Microsporidia MB* is found predominantly associated with *Anopheles gambiae* s.s and *Anopheles coluzzii* in Ghana

**DOI:** 10.1038/s41598-021-98268-2

**Published:** 2021-09-20

**Authors:** Jewelna Akorli, Esinam Abla Akorli, Seraphim Naa Afoley Tetteh, Godwin Kwame Amlalo, Millicent Opoku, Rebecca Pwalia, Michelle Adimazoya, Dorcas Atibilla, Sellase Pi-Bansa, Joseph Chabi, Samuel Kweku Dadzie

**Affiliations:** 1 Department of Parasitology, Noguchi Memorial Institute for Medical Research, University of Ghana, P. O. Box LG 581, Legon, Accra, Ghana; 2 Vestergaard-NMIMR Vector Labs, Noguchi Memorial Institute for Medical Research, University of Ghana, P. O. Box LG 581, Legon, Accra, Ghana; 3 Entomology Unit, Department of Clinical Laboratory, Kintampo Health Research Centre, P.O. Box 200, Kintampo, Ghana

**Keywords:** Microbial ecology, Entomology

## Abstract

A vertically transmitted microsporidian, *Microsporidia MB*, with the ability to disrupt *Plasmodium* development was reported in *Anopheles arabiensis* from Kenya, East Africa. To demonstrate its range of incidence, archived DNA samples from 7575 *Anopheles* mosquitoes collected from Ghana were screened. *MB* prevalence was observed at 1.8%. *An. gambiae* s.s constituted 87% of positive mosquitoes while the remaining were from *An. coluzzii*. Both sibling species had similar positivity rates (24% and 19%; *p* = 0.42) despite the significantly higher number of *An. gambiae* s.s analysed (*An. gambiae* s.s = 487; *An. coluzzii* = 94; *p* = 0.0005). The microsporidian was also more prevalent in emerged adults from field-collected larvae than field-caught adults (*p* < 0.0001) suggestive of an efficient vertical transmission and/or horizontal transfer among larvae. This is the first report of *Microsporidia MB* in *Anopheles* mosquitoes in West Africa. It indicates possible widespread among malaria vector species and warrants investigations into the symbiont’s diversity across sub-Saharan Africa.

## Introduction

Mosquitoes remain very important vectors of human disease, and several efforts are being made to reduce the burden they pose to human and animal health. As the progress of chemical-based vector control interventions is continually threatened, there is increased focus on alternative strategies including biotechnological ones for disease control^1^. Therefore, the search for natural mosquito-associated symbionts with the ability to reduce vector competence has been a growing interest.

It has recently been demonstrated that a vertically transmitted microsporidian prevalent in *An. arabiensis* in Kenya disrupts *Plasmodium* development^2^. Contrary to other mosquito-associated microsporidians, *Microsporidia MB* does not confer any significant negative effect on host’s fertility, fecundity, development, and longevity^2–4^. These characteristics make *Microsporidia MB* an appealing candidate for control of parasite transmission in *Anopheles* mosquitoes.

A recent discovery revealed the prevalence of *Microsporidia MB* only in *An. arabiensis*^2^. However, malaria is transmitted by a wide range of Anopheline species with different ecological ranges and varied efficiencies in transmitting *Plasmodium*^5,6^. In this study, we sought to extend the geographical range and identification of *Microsporidia MB* to other *Anopheles* species. We screened archived *Anopheles* mosquito DNA from field collections of several unrelated projects to detect the existence of *Microsporidia MB* among *Anopheles* populations in Ghana. To the best of our knowledge, this is the first study since the discovery of this microsporidian in *An. arabiensis* from Kenya that has investigated its incidence in different *Anopheles* mosquito species and from another geographically distant African population.

## Results

### *Microsporidia MB* detected in *An. gambiae* s.s and *An. coluzzii*

**Figure 1:**
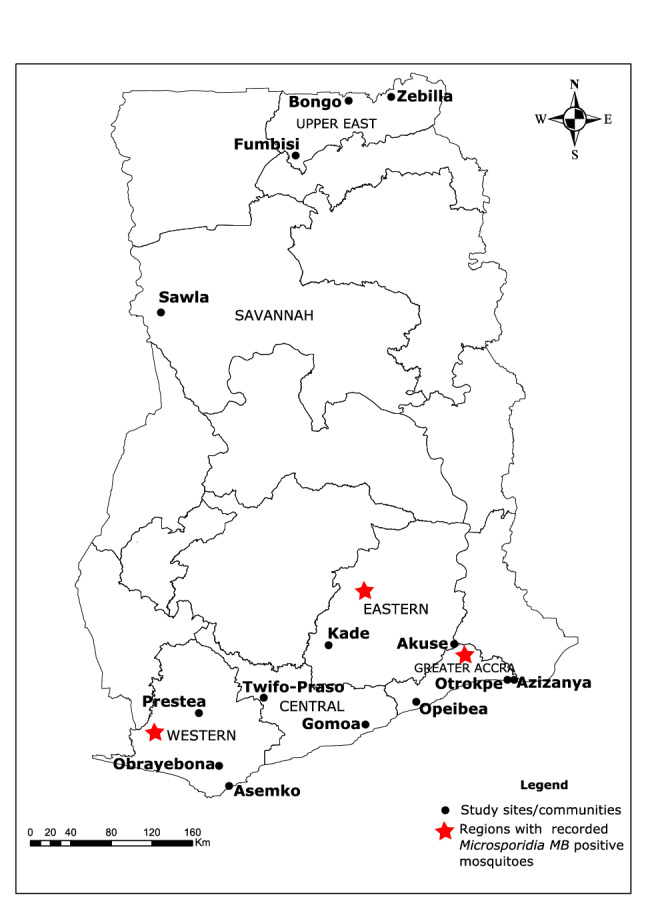
Map of Ghana showing study sites where mosquitoes were collected. Only the regions (6 out of 16) where the study sites are located are named in upper cases. Study sites are named in bold. Red stars depict regions where *MB*-positive mosquitoes were recorded. Map was created using ArcGIS v10.4.1.

**Figure 2:**
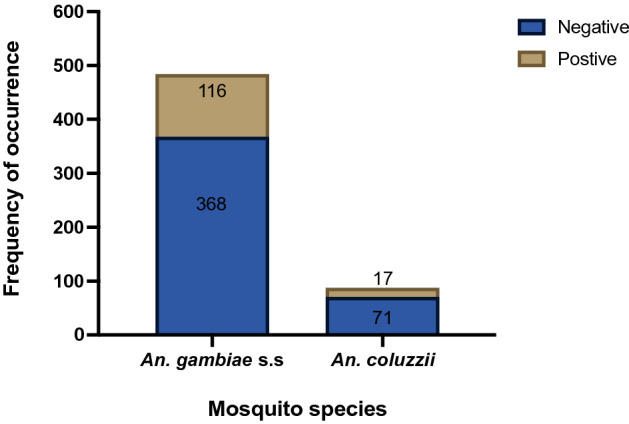
Microsporidia positivity rate in *An. gambiae* s.s and *An. coluzzii*. Numbers within the bar plot show the total number of mosquitoes observed for each status.

*Microsporidia MB* was detected in 133 out of 1158 DNA samples from mosquitoes collected across Ghana (Fig. 1). Accounting for the number of individual mosquitoes that went into pools for DNA extraction, a total of 7575 individual mosquitoes were involved in this analysis (Fig. S1). *MB* was found only in the DNA samples from single mosquitoes, giving an overall *MB* prevalence of 1.8% (133/7575) in the total number of mosquitoes analysed. *Microsporidia MB* was identified only in the DNA from *An. gambiae* s.s and *An. coluzzii*; the former constituting 87.2% of *MB*-positive mosquitoes (Fig. 2; Supplementary Table S1). The distribution of *MB* infection among *An. gambiae* s.s and *An. coluzzii* was 23.9% and 19.3%, respectively (Fig. 2) and this rate did not differ significantly between the two sibling species (χ^2^ = 0.66; *p* = 0.42).

### Higher *MB* detection rate in field-caught larvae than adults

The DNA samples used in this study comprised those extracted from adults that emerged from field-collected larvae and those that were collected from the field as adults. We were interested in establishing whether infections were more prevalent in newly emerged adults (from field-caught larvae) or in the field-caught adults. This was also to assess which of the two life stages would increase the probability of finding *MB* in a field study aimed at *MB* discovery. For this analysis, *An. arabiensis*, *An. melas* and *An. funestus* were excluded since they did not show positive infection. Of the remaining 7534 *An. gambiae* s.l mosquitoes, ~ 90% were field-caught adults. However, among the remaining 10% of samples that were initially collected from the field as larvae, 17.3% recorded positive for *MB*. This was significantly higher than the prevalence (0.13%) observed in field-caught adults (χ^2^ = 1092; *p* < 0.0001).

## Discussion

We make the first report of *Microsporidia MB* in *An. gambiae* s.s and *An. coluzzii* following identification of the symbiont in *An. arabiensis*. This does not only demonstrate the existence of the microsporidian in another predominant malaria vector species in Africa but also extends its incidence from East to West Africa. The prevalence of *MB*-positive mosquitoes was estimated to be 1.8%, which is within the rate of < 1–9% reported for *An. arabiensis*^2^. The present study took advantage of archived mosquito DNA samples which were either collected from the field as larvae or adults from different study sites over 5 years.

*Anopheles gambiae* s.s and *An. coluzzii* are the predominant malaria vectors in Ghana^7–9^. They are often found in sympatry with one species usually being more abundant^9,10^. Contrary to the study by Herren and colleagues^2^, a handful of *An. arabiensis* was analysed in the present study. *Anopheles arabiensis* is more commonly found in the arid north of Ghana where rainfall is observed within a few months in a year. In studies conducted in Ghana that are focused on *Anopheles* distribution, between 2–3% are *An. arabiensis* despite the collection of large numbers of mosquitoes^7,11^. However, we acknowledge that there were more collections from the south of the country, especially Greater Accra, which contributed 40% of the DNA samples used in this study and 89% of *MB*-positive mosquitoes. Further studies to investigate variations in mosquito species density and seasonal prevalence of *Microsporidia MB* will shed more light on the field dynamics of the symbiont in these mosquito populations.

DNA samples from mosquitoes initially collected as larvae from the field showed significantly higher *MB*-positivity than those collected as adults. Microsporidians can be transmitted both vertically and horizontally^12^. The efficiency with which *MB* is transmitted vertically depends on the intensity in the ovaries of the female parent. Horizontal transfer was initially speculated to occur in the larval habitat^2^ which would increase the spread of *MB* from few infected larvae to many in the breeding site, but this transmission route has recently been shown not to occur in larvae under laboratory conditions^13^. In effect, our detection of higher prevalence among newly emerged adult mosquitoes is more likely to be attributed to highly efficient vertical transmission from female parent to offspring^2^. In the laboratory, the intensity of *MB* in adult mosquitoes has been observed to increase with age^2^. However, in the wild where conditions are very dynamic, the effect of larval habitat conditions and adult age on *MB* intensity in mosquitoes may differ. For example, larval diet has been shown to affect the microbial composition of mosquitoes in their adult life stages^14^. It is also established that microbial diversity decreases in female adults mainly because of proliferation of certain bacteria species following sugar and/or blood feeding^15^. It, therefore, makes it challenging to explain variations in bacterial diversity among field-caught adult mosquitoes since their feeding histories are unknown^16^. Several environmental and physiological statuses could potentially affect the intensity of *MB* observed in adults and variations in infection prevalence could be governed by factors that are yet to be investigated. Given that collecting large numbers of adult mosquitoes in the field may prove more challenging than larval sampling, our results have also shown the increasing chances of finding *MB* infections in a population when larvae are collected in field studies^2^.

While the data presented here shows basic information about the ecological spread of *Microsporidia MB*, it has nonetheless demonstrated a potential widespread occurrence of *Microsporidia MB* among *Anopheles* mosquitoes across sub-Saharan Africa. It warrants further investigation of the diversity, environmental dynamics, and interactions with other mosquito symbionts for a clearer understanding of their possible use in malaria control.

## Methods

### Description of *Anopheles* samples

We retrieved *Anopheles* mosquito DNA samples from various studies that have been conducted at the Noguchi Memorial Institute for Medical Research, University of Ghana between 2014–2019. Mosquitoes had been collected from different sites across the country (Fig. 1) either as larvae or adults during the rainy season period. When collected as larvae, they were reared to adults for experimental assays before DNA was extracted. Most DNA samples were from single female mosquitoes while few were extracted from pools of 25 (Supplementary Table S1). The species information on the samples were retrieved from the different projects. Where the mosquitoes were only identified as *An. gambiae* s.l, these were further assessed for their sibling species identification using Restriction Fragment Length Polymorphism (RFLP)^17^ or SINE^18^ methods when they were positive for *Microsporidium MB* infection.

### Quality check of archived samples

Different extraction methods, including CTAB, Trizol RNA/DNA and columns had been used by the various projects to obtain DNA. It was therefore expected that the DNA integrity would differ among samples. The samples would have also gone through some freeze–thaw cycles which would compromise the quality of the DNA and result in false negatives. To address this concern, we randomly selected samples for DNA quantification with Qubit Fluorometer (Thermofisher, UK) and purity using the BioDrop (Biochrom, UK). Average DNA concentration was 34.8 ng/μL (1.02—204 ng/μ) and average purity was 1.7 (1.14–2.17). To reduce potential contaminants that would limit PCR, DNA samples were diluted 1 in 10.

### Detection of *Microsporidia MB*

A total of 1158 DNA samples were screened for *Microsporidia MB* using MB18SF/ MB18SR primers^2^ and with *Microsporidia MB* DNA obtained from Jeremy Herren’s lab as positive control in each set of reactions run. Diluted DNA samples were used in a first round of PCR reactions. The PCR included in the final reaction 1X One-Taq Master mix, 0.4 µM of each primer and 1µL of DNA template. The cycling conditions were as described in^2^. Products were loaded and run on a 2% agarose gel stained with SYBR Safe DNA stain (Invitrogen) and viewed under a blue-light transilluminator. The PCR reaction was repeated for samples that showed no band for *Microsporidia MB* using an increased volume of the diluted DNA and/or using 1µL of the stock DNA sample to confirm the initial results. The band size for *Microsporidia MB* detection was ~ 500 bp (Supplementary Fig. S2).

### Statistical analyses

Contingency analyses to compare independence observed positivity between mosquito species was performed using a two-sided Chi-squared test with Yate’s correction. The distribution of *An. gambiae* s.s and *An. coluzzii* from the study sites was tested with a two-tailed unpaired Mann–Whitney non-parametric test. All test significance was accepted a *p* < 0.05.

## Supplementary Information

Supplementary Information.
