# Supplementary material for: Microsporidia MB is found predominantly associated with Anopheles gambiae s.s and Anopheles coluzzii in Ghana
Source: Sci Rep. 2021 Sep 20;11:18658. doi: 10.1038/s41598-021-98268-2 (PMC8452686; doi:10.1038/s41598-021-98268-2)
Supplement: Supplementary file 1 — Supplementary Information. [file 41598_2021_98268_MOESM1_ESM.docx]

**Table S1: Details of the *Anopheles* mosquito DNA samples used in the study.** ‘nd’ designates ‘non determined’ and represents where prior molecular species information on *An. gambiae* s.s and *An. coluzzii* was not available from the previous study from which the DNA samples were retrieved. For these species, identification was performed only when DNA showed positive for *Microsporidia MB*.

**Fig S1: The total number of each member of *Anopheles gambiae* complex analysed.** The overall total number of mosquitoes involved in the analysis was N=7575.


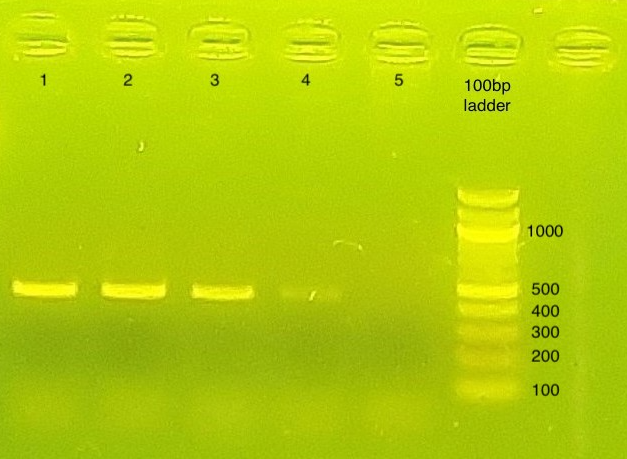


**Fig S2: Gel photo for quality check and band size estimation of *Microsporidia MB* positive control.** Well 1= mosquito DNA spiked with MB; 2= 1 in 10 diluted MB DNA; 3= 1 in 20 diluted MB DNA; 4= 1 in 100 diluted MB DNA; 5= no template control. 100bp ladder (NEB #N3231L).
